# Alzheimer’s disease: where do we stand now and what are the strategic interventions?

**DOI:** 10.3389/fncel.2025.1655342

**Published:** 2025-09-10

**Authors:** Andrea González, Stephanie Geywitz, Ricardo B. Maccioni

**Affiliations:** 1 International Center for Biomedicine (ICC), Santiago, Chile; 2 Laboratory of Neuroscience and Functional Medicine, Faculty of Science, University of Chile, Santiago, Chile

**Keywords:** Alzheimer’s disease, etiopathology, immunomodulation, prevention, early detection diagnosis, nutraceuticals

## Abstract

Alzheimer’s disease (AD) is a multifactorial neurodegenerative disease, the primary cause of dementia in people over 65 years old. AD is characterized by two molecular hallmarks, the intracellular neurofibrillary tangles of tau and amyloid beta oligomers, which are aggregates of hyperphosphorylated tau and amyloid beta peptides, respectively. These hallmarks gave rise to the two main theories that have opened the way for available treatments, such as FDA-approved memantine, and Aβ (aducanumab, lecanemab) and tau immunotherapies. Tau immunotherapy, especially multitarget approaches, has been recently proven effective. However, drugs against amyloid plaques had a non-successful outcome, despite their contributions to AD knowledge. An innovative approach comes from the multitarget concept, based on bioactive molecules and nutraceuticals. Interestingly, the use of early detection biomarkers such as Alz-Tau^®^, SIMOA^®^, and the recent Lumipulse™ test, are an important support to orient AD therapies based on the modifications of the styles of life. This includes physical exercise, a healthy diet, mindfulness, and cognitive stimulation, among others. All of the above analyses are critical to switch the focus to the prevention of AD.

## Graphical Abstract

**Figure:**
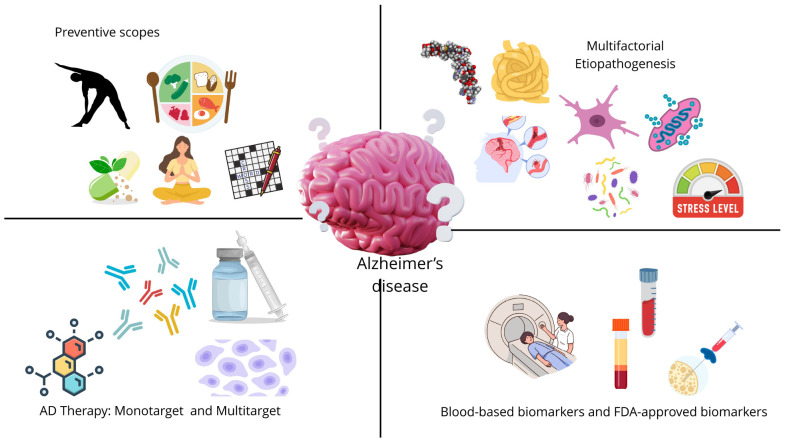
Alzheimer’s disease current therapies, detection strategies, preventive scopes and etiopathogenesis summarized in this review.

## Introduction

Alzheimer’s disease is currently the leading cause of dementia worldwide in elderly adults older than 65 years old (Alzheimer Association, 2024). Indeed, dementia is currently the third leading cause of death in the United States. Due to the accelerating aging in the population, its understanding and management represent a significant challenge in contemporary medicine, particularly given its status as the most prevalent cause of dementia in individuals over the age of 65 (Weninger et al., 2016; Alzheimer Association, 2024). In a WHO report, in 2022, 50 million people were affected by dementia worldwide, and it’s projected that by 2050, 150 million people will be affected (Kerwin et al., 2022). AD also constitutes a major puzzle for the world community, considering the enormous social and economic impacts on families and caregivers, as well as on the economies of the countries. This complex, multifactorial neurodegenerative disorder elicits a gradual decline in both cognitive and non-cognitive functions, generating a substantial burden on patients, their families, and broader society (Jain and Sharma, 2021). The molecular markers of Alzheimer’s disease pathology are classically defined by the presence of extracellular amyloid plaques, primarily composed of aggregated amyloid-beta peptides, and intracellular neurofibrillary tangles, which consist of hyperphosphorylated tau protein (Brion, 1998; Blennow et al., 2006; González et al., 2022b). The initial identification of these neuropathological hallmarks by Alois Alzheimer in 1906 has led to the formulation of prominent yet contested theories regarding the disease’s etiology (Trejo-Lopez et al., 2022). The Amyloid Cascade Hypothesis postulates that the accumulation and aggregation of amyloid-beta peptides are the primary drivers of the disease, initiating a cascade of events that ultimately lead to neuronal dysfunction and death (Sadigh-Eteghad et al., 2015). Conversely, the Tau Hypothesis establishes that abnormalities in tau protein, such as hyperphosphorylation and subsequent aggregation into neurofibrillary tangles, are the central pathogenic events in Alzheimer’s disease (Maccioni et al., 2009, 2010; Crespo-Biel et al., 2012). Despite the extensive research efforts directed toward these two prominent hypotheses, therapeutic strategies targeting either amyloid-beta or tau have yielded limited success, underscoring the complex and multifactorial nature of Alzheimer’s disease (González et al., 2023).

Currently, only palliative treatments and post-clinical FDA-approved biomarkers are available in the clinic. Why have all the previous treatments failed to improve the quality of life of the patient and, consequently, of their caregivers? To answer that question, it is required to go to the multifactorial etiology of AD (González et al., 2023). This disease goes beyond its major molecular hallmarks, the neurofibrillary tangles and the amyloid plaques. Several damage signals trigger the activation of the microglia, promoting neuroinflammation (Singh, 2022; Wang C. et al., 2023). This promotes a chronic pro-inflammatory microenvironment, which leads to neurodegeneration (Kinney et al., 2018). Current FDA-approved therapies, such as memantine (Parsons et al., 2013) and others, only act on one target, which would explain in part why the improvement of cognitive performance is not satisfactory. Now, novel therapies focus on multitarget strategies, usually combining nutraceuticals, bioactive compounds, such as Andean shilajit (Andrade et al., 2023), and a healthy diet. This has proven to be the best approach so far.

## The etiopathogenesis of AD: Aβ, tau, or both?

In 1901, Alois Alzheimer described for the first time a case of a 50 years-old woman, Auguste Deter, the first reported case of Alzheimer’s disease. In the report, he described that Auguste had several memory issues, disorientation, and hallucinations, among other psychiatric symptoms. After passing in 1906, Dr. Alzheimer performed histological studies on her postmortem brain (Stelzmann et al., 1995). This allowed him to discover two abnormalities present in Auguste’s brain: amyloid plaques between the neurons and neurofibrillary tangles inside the neurons. This discovery established the two main hallmarks of AD: extracellular amyloid plaques, constituted by Aβ peptide, and neurofibrillary tangles, constituted by hyperphosphorylated tau protein (Roda et al., 2022; Abyadeh et al., 2024). The accumulation of both amyloid peptide 1–42 and hyperphosphorylated tau protein generates oligomers (AβO and TauO, respectively), leading to a disruption of neuronal function and exerting a toxic influence on the brain, since they trigger the misfolding of adjacent proteins into aggregates or oligomers (Mroczko et al., 2019). However, the debate about which one is the principal contributor to the development and progression of AD has extended to the present day.

Current evidence states that regarding the amyloid plaques, which are constituted by AβO, these are also present in cognitively healthy elders (Rodrigue et al., 2012), in some cases even more than AD patients. Also, cognitive decline, the main symptom of AD, is not directly correlated with the amyloid-plaque burden in the brain, a reason why the current amyloid therapies had failed (Haass and Selkoe, 2022). Despite those facts, new evidence has shed light on the involvement of some soluble Aβ fragments, among several other signals, which can be recognized by the microglia as a danger signal, and not the amyloid plaque as initially thought (Lučiūnaitė et al., 2020). The latter is supported by other evidence, demonstrating that through the gut-brain axis, some bacteria can secrete amyloid-like peptides and activate Alzheimer’s disease pathways in a neuronal cell line (Blanco-Míguez et al., 2021).

**FIGURE 1:**
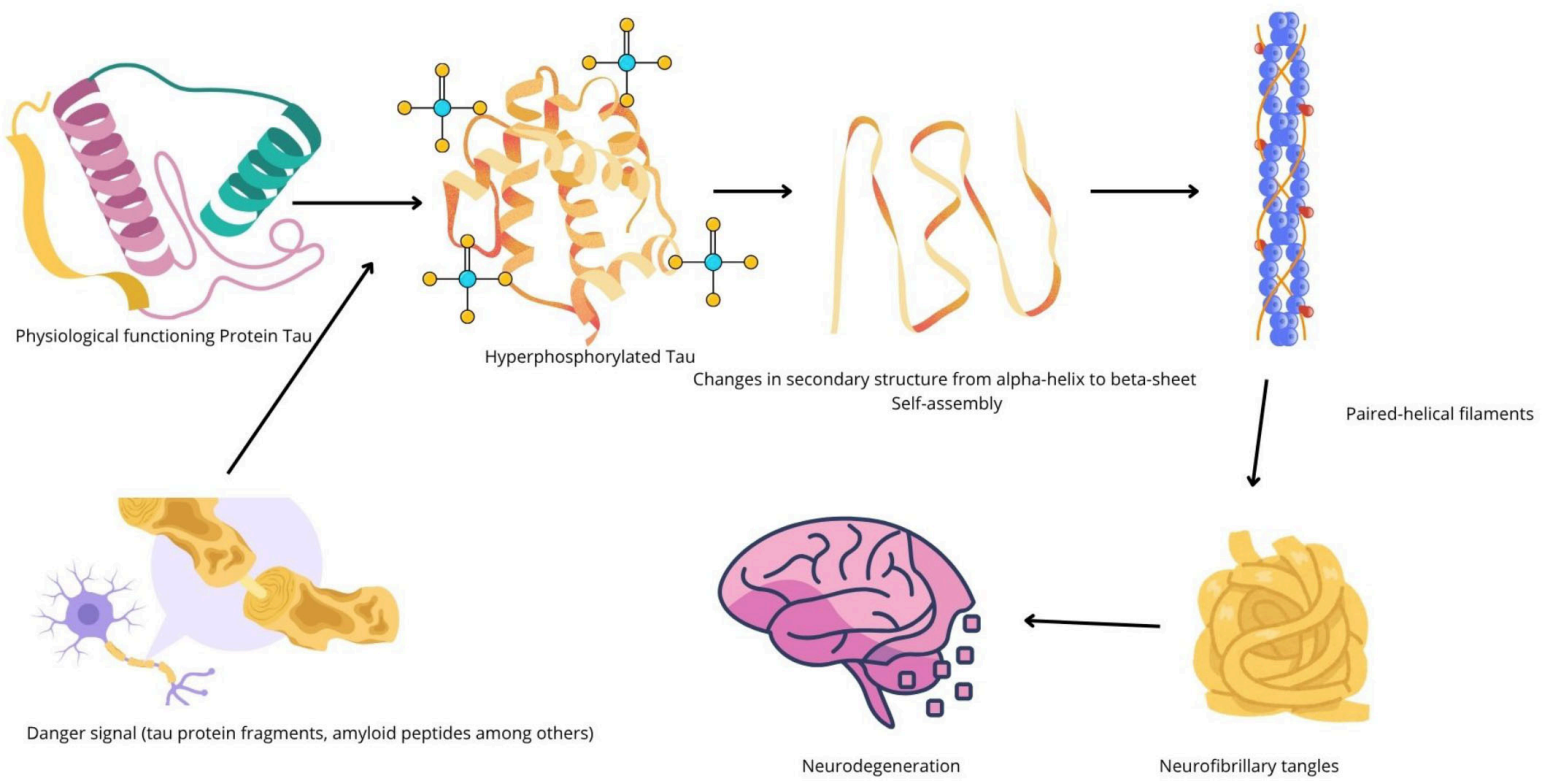
Schematic representation of tau protein aggregation in Alzheimer’s disease. Conformational changes due to hyperphosphorylation of tau leads to pathologic tau self-assembly, and the formation of neurofibrillary tangles, one among the main hallmarks of Alzheimer’s disease (AD).

Neurofibrillary tangles, on the other hand, are constituted by TauO. Tau is a microtubule-associated protein (MAP) whose main function is to direct the formation of microtubules that allow the formation of dendrites in neurons (Crespo-Biel et al., 2012). Hyperphosphorylation of tau protein leads to conformational structure changes, from an α-helix to a β-sheet, allowing their self-assembly into pair-helical filaments (PHF) and later, neurofibrillary tangles (Luna-Muñoz et al., 2007; González et al., 2022b). It has been demonstrated that, contrary to amyloid plaques, hyperphosphorylated tau protein, and neurofibrillary tangles correlate well with cognitive decline and brain atrophy (Maccioni et al., 2006; Slachevsky et al., 2016). This process is illustrated on Figure 1. Thus, tau protein has now emerged as a novel candidate to conduct further research regarding novel therapies for AD and the development of early detection biomarkers, two milestones required to promote AD prevention and the slowing of the onset of cognitive symptoms.

## Integration of several onset factors and new theories: neuroinflammation as the key feature

To understand AD, it is key to identify it as a multifactorial neurodegenerative disease. Several signals can trigger the onset and are also involved in the progression of the disease.

Two of the principal signals were discussed above, as the two main hallmarks in AD: amyloid plaques and neurofibrillary tangles. However, several other contributors either to the onset or the progression of AD can be mentioned:(i) Metabolic Dysfunction: This is perhaps one of the major contributors to metabolic issues, such as AD, alongside the main hallmarks. Evidence supports that AD brains have several lower expression of glucose transporters (Kalaria and Harik, 1989; Liu et al., 2008) and brain insulin resistance (Bosco et al., 2011; Kellar and Craft, 2020), just to mention a few. Also, it should be considered that patients with type 2 diabetes have 2.5 times higher risk of developing AD, and also AD patients have two times higher risk of developing type 2 diabetes (Sebastião et al., 2014; Mushtaq et al., 2015). In that regard, AD is now considered a novel type 3 diabetes (González et al., 2022a); (ii) Gut-brain axis and commensal bacteria: several studies sustain that AD patients suffer changes in the intestinal microbiota, promoting pro-inflammatory signals that reach the brain and trigger pro-inflammatory signals (Jiang et al., 2017; Varesi et al., 2022). Some of these bacteria can secrete amyloid-like peptides that can activate the microglia, in agreement with one of the original hypotheses. Studies on germ-free 3xTg mice model showed a significant reduction in amyloid plaques and neurofibrillary tangles as compared to the 3xTg control (Chen et al., 2022). Also, the proinflammatory pathway C/EBPβ/AEP, associated with polyunsaturated fatty acids (PUFA), is downregulated in these germ-free 3xTg mice as compared to the 3xTg control (Chen et al., 2022). Thus, gut microbiome regulates AD and associated cognitive disorders via PUFA-mediated neuroinflammation; (iii) Mitochondrial dysfunction: Emerging as a novel theory, mitochondrial dysfunction is now considered one of the relevant features in AD. In the brain, neurons require a high amount of energy to maintain synaptic function and plasticity (Reiss et al., 2024), and mitochondrial dysfunction is among the first detectable changes in AD. Mitochondrial dysfunction in AD compromises neuronal function and viability, contributing to the onset of AD symptoms due to early neuronal death (Reiss et al., 2024). This is closely related to other factors, such as metabolic dysregulation, calcium homeostasis disruption, oxidative stress and mitochondrial quality control impairment, all observed in AD (Jayatunga et al., 2020); (iv) Infections: it has been reported that viruses and bacteria that can cross the blood-brain barrier are related to cognitive decline and neuronal death, such as Herpes viruses (Deatly et al., 1990). Notably, a recent study demonstrated that vaccination against the herpes-zoster virus decreased dementia in elderly adults (Eyting et al., 2023); (v) Vascular dysfunction: As brain function depends on continuous delivery of oxygen and energy substrates, such as glucose, a suitable cerebral vasculature that allows these elements is required (Hirsch et al., 2012). The regional cerebral blood flow (rCBF) is tightly regulated for this purpose. As part of the cerebral vasculature, the blood-brain barrier (BBB) regulates the passage of oxygen and nutrients and the removal of metabolic waste products. It also prevents entry of plasma constituents and protects the brain from infection (Zhao et al., 2015). In AD, vascular lesions such as arteriolosclerosis, microinfarcts, hemorrhage, atherosclerosis, and cerebral amyloid angiopathy are prevalent in 80% of cases diagnosed with AD (Toledo et al., 2013), all the later associated with a decrease in brain microcirculation (de la Torre and Mussivand, 1993). These lesions lead to a reduction in the rCBF. This hypoperfusion is attributed to an impaired vascular regulation by soluble A (Dietrich et al., 2010). This peptide is vasoactive and constricts arterioles. Also, it has been demonstrated that Aβ oligomers constrict capillaries (Nortley et al., 2019). This allowed to propose that vascular dysfunction could lead to AD (de la Torre and Mussivand, 1993).

All of the above-mentioned onset signals have a common feature: the trigger of pro-inflammatory signals that activate microglia and promote a pro-inflammatory microenvironment in the brain (Rubio-Perez and Morillas-Ruiz, 2012; Twarowski and Herbet, 2023).

The latter is summarized in the neuroimmunomodulation theory (Maccioni et al., 2009, 2010), which states that it is a cyclic event, where these pro-inflammatory signals (especially tau-related signals) (Morales et al., 2013) activate the microglia and pro-inflammatory cytokines, such as IL-6 and TNF-α (Zheng et al., 2016; Culjak et al., 2020), activate downstream signaling pathways, such as the one mediated by NFkβ and promote upregulation of key proteins, such as the kinases CDk5 and GSK3β (Zheng et al., 2005; Kimura et al., 2014; Saito et al., 2019). This upregulation, in turn, leads to hyperphosphorylation of tau protein, which leads to the conformational changes that promote self-assembly, leading to the formation of paired-helical filaments and neurofibrillary tangles that eventually lead to neurodegeneration (González et al., 2022b). Fragments of these neurofibrillary tangles can act as signals for the activation of another microglia; thus, the cycle continues.

**FIGURE 2:**
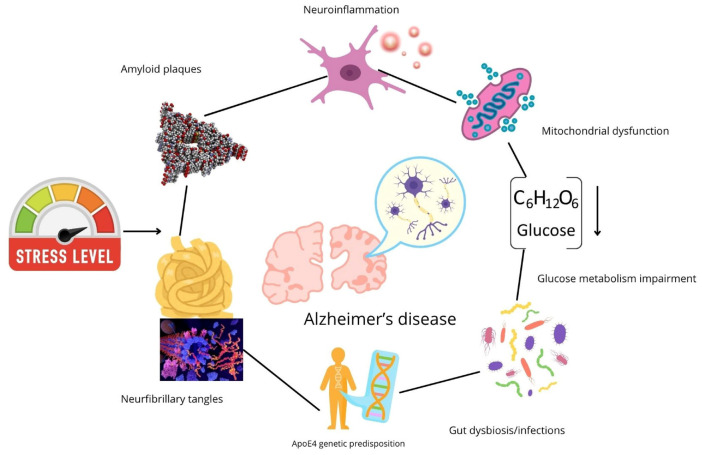
Alzheimer’s disease etiopathogenesis. Beyond the classic molecular hallmarks of Alzheimer’s disease (AD), the neurofibrillary tangles and amyloid oligomers, other factors contribute to the onset and progression of this disease. Among them, mitochondrial dysfunction, oxidative stress, gut dysbiosis, infections, genetic predisposition (APoE4 allele), glucose metabolism impairment, among others. This is why Alzheimer’s disease is a multifactorial pathology.

As we come to terms with the multifactorial nature of AD, summarized in Figure 2, it is no wonder that current therapeutic approaches have already failed.

## Stress and the onset of Alzheimer’s disease

Human beings are viewed holistically as biopsychosocial individuals, with determining factors in their lives such as biological, genetic, chronological, and environmental factors. Environmental factors include stressors, which are one of the many causes of neurodegenerative diseases, including AD (Doyle et al., 2014; Stuart and Padgett, 2020).

Stress affects people of all genders and ages and varies according to each individual’s stressful experiences. When a person constantly faces stressors, physical and chemical changes occur that affect their health. Stress allows people to cope with the obstacles they encounter, and therefore, each individual will respond differently, assessing their ability to cope. Stress can be chronic, depending on the duration and intensity of the aversive event. Changes occur in the nervous system (CNS), which produce alterations in the entire organism, that will be expressed later as multiple disorders. When there are sudden changes in the nervous system, in order to maintain homeostasis, it is forced to demand compensation from the various systems, resulting in the overload of activity without control, and finally with consequences that primarily affect the proper functioning of the brain (Vallejo-Johnson and Marcial-Velastegui, 2018).

We understand that stress is a natural human response to situations of extreme emotional tension. It manifests itself in a state of intolerance and irritability in response to the life situation experienced. Cannon points out that the body reacts to threats by activating two systems: the sympathetic nervous system and the endocrine system (Cannon, 1932). When activated, the body returns to calm.

However, prolonged stress causes excess production of adrenaline, noradrenaline, glucocorticoids, and cortisol, which disrupt the body’s homeostasis process. This affects the neurons of the hippocampus, generating progressive neuronal loss that impairs activities of daily living, as reflected in AD. Lifestyles focused on physical exercise, cognitive stimulation, and a healthy diet can promote a more favorable adaptive response for emotional wellbeing and the homeostasis process the body faces in times of stress.

It is important to recognize stress as a major factor in AD pathogenesis, as it correlates with the onset of depressive symptoms or stress-related pathologies. It is worth noting that these stress-related pathologies could promote AD-type neurodegenerative disorders (García et al., 2012).

Brain structures such as the hippocampus, amygdala, striatum, and cortex are actively involved in processing information associated with learning and memory. These cognitive processes induce changes in synaptic plasticity under normal conditions, but in patients or in transgenic animal models, these processes are affected by the development of AD type cognitive decline. In transgenic models of AD, the way this pathology affects synaptic plasticity has been studied. This work has been conducted at three levels of analysis: molecular, cellular, and cognitive-behavioral. There are behavioral tasks that induce a high release of stress hormones, which can affect learning and memory consolidation. AD can impair motor performance because certain tasks require good motor performance, reflecting cognitive impairment (Bello-Medina et al., 2022).

## AD biomarkers: in need of an early detection

The shortcomings of current monotarget therapeutic approaches highlight the critical need for early detection biomarkers that can identify individuals at risk of developing Alzheimer’s disease, potentially enabling preventative strategies or interventions that can delay or even halt the onset of clinical symptoms. However, current alternatives only include detection when the clinical symptoms are evident. Some of the current detection biomarkers for AD are:

(i)    Neuroimages: This includes nuclear magnetic resonance (NMR) and positron-emission tomography (PET-scan) as the most relevant ones. These techniques involve computerized images. NMR imaging uses radio waves and strong magnetic fields to create detailed images of organs and tissues inside the body, in this case, the brain. Usually, brain atrophy is observed with this technique. PET-SCAN, on the other hand, uses fluorescent or radioactive tracers to visualize and measure metabolic processes and other physiological activities in the body, such as fluoro-deoxy-glucose. The FDA has approved PET-scan imaging with an amyloid-beta and tau probes (Chandra et al., 2019; Afzal et al., 2021; Maschio and Ni, 2022; Wang R. et al., 2023), the most recent ones directed to tau as biomarkers for AD (Xia et al., 2013; Kolb and Andrés, 2017). Nevertheless, the main disadvantage is that this technology is expensive and only provides diagnosis once the cognitive decline is evident.
(ii)    Cerebrospinal fluid (CSF) biomarkers: The other FDA-approved biomarkers for AD are those evaluated in CSF (Maccioni et al., 2006). Several antibodies are employed to detect either amyloid-beta, tau, and, more recently, phospho-tau, in CSF samples (Mattsson et al., 2017; Kiđemet-Piskač et al., 2018; Barthélemy et al., 2020; McGrowder et al., 2021). These biomarkers can be evaluated using several techniques, with the most commonly used being an ELISA (enzyme-linked immunosorbent assay), according to the National Institutes of Health (NIH). Nonetheless, CSF sampling is an invasive procedure that requires trained personnel. Also, since it provides a diagnosis after the manifestation of cognitive decline symptoms, it is not useful for early detection.

While neuroimaging techniques such as PET scans and cerebrospinal fluid analysis for biomarkers like tau, phosphorylated tau, and amyloid-beta peptide are employed for diagnosis, their limitations in providing early detection and their invasive nature require the development of more accessible and sensitive diagnostic tools. Thus, current FDA-approved biomarkers for AD do not allow early detection. Added to that, the costs (over US$1000 for PET-SCANS and US$650 for CSF tests) and accessibility to these tests are a severe limitation for a routine-based implementation in low-income countries, which exhibit the highest rate of increase in AD. Thus, at present, efforts are leading to the implementation of cost-effective biomarkers that provide early detection, which allows screening of patients before the manifestation of cognitive decline.

(iii)    Blood-based biomarkers: Due to the lack of early detection biomarkers, efforts have been made to develop cost-effective early detection biomarkers, which must be accessible and less invasive for the patients (Brickman et al., 2021; Ashton et al., 2023b). In this regard, several blood-based biomarkers are evaluated, in which we can highlight (a) The Alz-tau biomarker: validated by four clinical trials, this novel blood-based biomarker allows screening of pre-clinical populations at risk of developing AD. It is based on the detection of platelet tau variants, including high-molecular-weight (HMW) and low-molecular-weight (LMW) forms (Guzmán-Martínez et al., 2019), using a novel tau-51 monoclonal antibody (González et al., 2020) in a western blot analysis. The HMW/LMW ratio correlates with brain atrophy and cognitive decline and allows screening of presymptomatic subjects. Currently, it is implemented in several hospitals and clinical laboratories; (b) Ultrasensitive SIMOA^®^ assay detection: another high-end novel biomarker is based on a novel SIMOA^®^ assay, which allows ultrasensitive detection of tau protein in serum and also CSF, at levels of pg/ml (Bayoumy et al., 2021; Ashton et al., 2023a). This novel technology is used for detecting and quantifying specific biomolecules, primarily proteins, in various biological samples. It is based on the isolation of individual immunocomplexes within femtoliter-sized reaction chambers, enabling the detection and counting of single molecules. This approach allows SIMOA^®^ to detect biomarkers at concentrations up to 1,000 times lower than traditional ELISA methods, often reaching the femtomolar (fg/mL) range. Currently, two of these SIMOA^®^ platforms, which detect ptau181 and ptau217, were recognized by the FDA as a breakthrough device due to the test’s potential for more effective diagnosis of AD; (c) Lumipulse technology: This technique uses chemiluminescent enzyme immunoassay (CLEIA) to measure biomarkers in bodily fluids, like blood or cerebrospinal fluid, for various diagnostic purposes. This technology has been evaluated in Alzheimer’s disease (Bayoumy et al., 2021; Keshavan et al., 2021; Gobom et al., 2022). Recently, one of these platforms, Lumipulse G pTau217/ß-Amyloid 1-42, which evaluates the plasma ratio between ptau217 and amyloid 1-42, received a breakthrough device recognition by the FDA.

Additionally, other biomarkers are being tested, such as those using mass spectrometry (Cilento et al., 2019). One of them, PrecivityAD2, which generates a ptau-217 and aβ 42/40 ratio, was clinically validated (Meyer et al., 2024). However, all of them require to be validated by neuropsychological tests to confirm the diagnosis.

This reflects the current advances in the search of early detection biomarkers, which is ongoing. Some of the former mentioned tests are being clinically implemented but not as a routine-based test, while others are still on clinical validation trials.

## Therapeutic approaches for AD: monotarget, multitarget and novel approaches

Current therapeutic interventions approved by regulatory agencies such as the FDA offer only symptomatic relief or aim to slow the progression of cognitive decline, without addressing the underlying disease mechanism (Wang Y. et al., 2023). The multifactorial nature of AD can explain, at least in part, why several therapeutic approaches failed. In this regard, current therapies for AD can be subdivided into two:

(a)    Monotarget therapies: These therapies aim at a single pharmacological target. The FDA-approved drugs are classified as monotarget, such as memantine, a cholinesterase inhibitor (Parsons et al., 2013; Tang et al., 2023). Novel current therapies include monoclonal antibodies that act against amyloid-beta have also been approved by the FDA: aducanumab, lecanemab, and donanemab (Haddad et al., 2022; PR Newswire, 2023; Söderberg et al., 2023). Currently, aducanumab is on phase IV clinical trial, and lecanemab in 2023 was approved by the FDA. However, as discussed above, amyloid beta does not have a direct relation with cognitive impairment in AD, not the way tau protein does. Which is why the majority (if not all) of the therapeutic approaches based on amyloid-beta have failed, due to lack of efficiency and some severe side-effects (Knopman et al., 2021; Atwood and Perry, 2023; Kurkinen, 2023). That is why novel therapies that target tau protein. In a recent study, a novel tau monoclonal antibody, specific for insoluble tau, employed as a therapeutic approach, improved tau pathology through the cytosolic antibody receptor TRIM21(Mukadam et al., 2023). The limitations of mono-target therapeutic approaches in Alzheimer’s disease have prompted a shift toward multi-targeting strategies that address multiple pathological pathways simultaneously (Maccioni et al., 2020).
(b)    Multitarget therapies: These multi-targeted approaches may involve the combination of components targeting different aspects of the disease, such as amyloid-beta production, tau phosphorylation, neuroinflammation, and oxidative stress. In this group, we can find all bioactive compounds, nutraceuticals, and functional foods that aim at more than one target. Some examples are nutraceutical formulations such as Brain-Up10^®^ or Durabrain™ in United States, multicomponent with several bioactive molecules containing Andean *shilajit* and vitamin B complex (Cornejo et al., 2011; Carrasco-Gallardo et al., 2012a; Guzman-Martinez et al., 2021b), which is supported by clinical trials. Then, we have bioactive compounds such as curcumin (Tang et al., 2017; Voulgaropoulou et al., 2019) and quercetin (Zaplatic et al., 2019; Khan et al., 2020), and functional foods such as berries (Subash et al., 2014), which are rich in anthocyanins (Sohanaki et al., 2016; Ma et al., 2018) and polyphenols (Reddy et al., 2020; Caruso et al., 2022; Li et al., 2023). This approach has been proven to be the most effective therapy to delay the progression and improve cognitive performance in AD.

It is worth mentioning that in the context of AD therapy, other novel approaches are:

(c)    Immunotherapy: This could be summarized in (i)Active immunotherapy: anti-Aβ vaccines, such as AN1792, the first one to be tested clinically (Thatte, 2001). However, during phase IIA, 6% of the patients developed meningoencephalitis due to an excessive Th-1-mediated inflammation. Currently, UB311 is on phase II clinical trial. This vaccine is composed by two synthetic T-helper peptide epitopes linked to Aβ 1-14 and possess safety vaccine designs and a delivery mechanism that increases Th-2 response (Wang et al., 2017). AADVac1, on the other hand is a tau epitope-based vaccine which induces specific antibodies targeting 3 or 4 conformational epitopes (Novak et al., 2019). AADVac1 treatment resulted in less brain atrophy and reduced cognitive impairment in a Phase I clinical trial. (ii) Passive immunotherapy: This therapy involves passive administration of monoclonal antibodies generated against the target protein, in this case, Aβ 1-42. Some of the monoclonal antibodies employed for Aβ- based immunotherapy are lecanemab and aducanumab, which are currently going on a phase II/III clinical trials (Thatte, 2001). Even though aducanumab was approved in 2021 by the FDA, a new phase II clinical trial is required to evaluate safety and efficiency as vasogenic edema was developed in trials (Arndt et al., 2018).

Anti-tau-based immunotherapy includes Zagotenemab, Tilavonemab, and bepranemab among others (Thatte, 2001). Semorinemab can bind to the six human tau isoforms and protect neurons, and a study in patients with moderate AD was performed (Ayalon et al., 2021). Phase 2 clinical trial of Zagotenemab showed increase in ptau181 and adverse effects with no efficiency (Fleisher et al., 2024). Tilavonemab was discontinued due to the lack of efficiency (West et al., 2017).

All the efficiencies and safety were compared in a recent study by Cai et al. (2025) (Ahmad et al., 2024). This study showed that Semorinemab was more effective in terms of cognitive decline prevention.

(d)    Stem cell therapy: A novel potential therapy worth to be explored is the stem cell therapy for AD. Four types of stem cells are available: embryonic stem cells, induced pluripotent stem cells, neural stem cells (NSCs) and mesenchymal stem cells (Ahmad and Sachdeva, 2022). Using AD rodent models, it was demonstrated that NSCs reduce tau and Aβ expression levels (Lee et al., 2015); promote neurogenesis and synapse formation (Ager et al., 2015; Lilja et al., 2015), reduce neuroinflammation (Zhang et al., 2016); and reverse cognitive deficits (Ager et al., 2015; Lilja et al., 2015; Zhang et al., 2016). Several clinical trials of stem cell therapy are ongoing (Ahmad et al., 2024), in Phase I and II. And more recently, a phase II study with laromestrocel demonstrated its efficiency and safety, as at 39 weeks post-treatment, it significantly reduced hippocampal atrophy, which correlated with an improvement in cognitive performance evaluated by MMSE (Rash et al., 2025).

## AD prevention: the five major tips to prevent and slow-down its progression

All the former therapeutic approaches and early detection are key for AD prevention or to slow down its progression. It is known that sporadic AD can be decreased if we modify our lifestyle (Guzman-Martinez et al., 2021a; González-Madrid et al., 2023), aligning at least with these major factors:

(i)    Exercise: Sedentary life is a risk factor for the development of AD; thus, exercise is a key player in AD prevention, especially aerobic exercise (Morris et al., 2017; Meng et al., 2020; López-Ortiz et al., 2021). It has been demonstrated that exercise stimulates the secretion of a neuroprotective hormone, irisin (Jin et al., 2018), which promotes the downregulation of the ERK-STAT3 pathway through the release of neprylisin (Kim et al., 2023). It was demonstrated that irisin rescues synaptic plasticity alongside the FNDC5 protein, which is associated with neuroplasticity, in an exercise-linked manner on Alzheimer’s disease mouse models (Lourenco et al., 2019). This is consistent with other studies that presented correction in memory deficits in mouse models of AD (Lourenco et al., 2017) and protective pathways in rat hippocampus (Lourenco et al., 2022). On the other hand, exercise also promotes the increase of brain-derived neurotrophic factor (BDNF) in people with multiple sclerosis (Shobeiri et al., 2022). This is also consistent with the stimulation of adult hippocampal neurogenesis (AHN) and the increase of BDNF in 5xFAD mice (Choi et al., 2018). Thus, within neurodegenerative diseases, such as AD and multiple sclerosis, exercise has neuroprotective effects (Mahalakshmi et al., 2020).
(ii)    Healthy diet: a diet rich in antioxidants, such as the Mediterranean diet, rich in vegetables, fruits, whole grains, olive oil, beans, and fish, is pivotal to prevent AD (Solch et al., 2022). Also, it helps to keep balance in the gut microbiota (Solch et al., 2022; Dissanayaka et al., 2024), since a gut dysbiosis generates damage signals, such as LPS and amyloid-like peptides, that promote neuroinflammation (Leblhuber et al., 2021), generally associated with a proinflammatory diet (Shi et al., 2023). Consistent with the latter, the Mediterranean diet improved main cognitive functions in AD patients (de la Rubia Ortí et al., 2018). Another example is the ketogenic diet, which is high-fat, moderate-protein, very-low-carbohydrate eating plan that induces a state of ketosis in the body (Lange et al., 2017). This opens new avenues in the treatment of Alzheimer’s disease, since this diet can effectively reduce the accumulation of amyloid-beta and tau proteins (Oliveira et al., 2024), reduce neuroinflammation (Xu et al., 2022), stimulate synaptic plasticity (Di Lucente et al., 2024) and modulate the gut microbiome (Dilmore et al., 2023) among other effects.
(iii)    Meditation/mindfulness: Meditation may offer promising benefits for individuals with Alzheimer’s disease (AD) and mild cognitive impairment (MCI), potentially slowing cognitive decline and improving well-being, as well as promoting AD prevention (Khalsa, 2015; Chen et al., 2020; Guzman-Martinez et al., 2021a). Meditation improves cognitive function in adults with cognitive decline (Innes et al., 2017). Also, it improves the blood flow to the brain (Khalsa et al., 2009), and promotes structural changes in patients with MCI or AD (Dwivedi et al., 2021), which includes a reduction of brain atrophy. This in turn improves brain connectivity. Also, meditation downregulates stress and anxiety, some of the most common neuropsychological symptoms in AD (Khalsa, 2015).
(iv)    Cognitive stimulation: solving puzzles, reading, and writing by hand promote cognitive stimulation, which in preclinical AD is associated with brain structure and cognitive function (Schultz et al., 2015). Studies have shown that in AD patients undergoing cognitive stimulation programs improve their cognitive plasticity (Zamarrón Cassinello et al., 2008) and in early-stage AD, it improves neuropsychological symptoms such as apathy (Buettner et al., 2011). Also, cognitive stimulation improves connectivity and cognition in AD patients (Behfar et al., 2023). Indeed, multisensory stimulation had a positive effect not only on cognition but also decreased depression and anxiety levels, thus improving neuropsychological symptoms on AD patients (Ozdemir and Akdemir, 2009).
(v)    Nutraceuticals and supplements: In addition to a healthy diet, it is important to complement it with relevant nutraceuticals and supplements that could benefit cognitive health and preserve brain function. A nutraceutical formulation, Brain-Up 10^®^, containing Andean shilajit and Vitamin B complex (Carrasco-Gallardo et al., 2012a), has been validated in clinical trials (Guzman-Martinez et al., 2021b). The Andean shilajit is rich in humic and fulvic acids, compounds that promote disassembly of tau fibrils *in vitro* (Cornejo et al., 2011). This formulation improves neuropsychological symptoms in patients, especially apathy (Guzman-Martinez et al., 2021b). In a pre-clinical study, the shilajit and Brain-Up 10^®^ formulation increased the number of neuronal processes and their length (Carrasco-Gallardo et al., 2012b). When the Andean shilajit was chemically fractionated, a neuritogenic effect *in vitro* of the fractions was observed, which was higher than that of the shilajit (Andrade et al., 2023). Regarding functional compounds, we can mention several, such as polyphenols (Choi et al., 2012; Caruso et al., 2022), flavonoids (Hasan et al., 2023), anthocyanins (Ma et al., 2018; Afzal et al., 2019), quercetin (Zaplatic et al., 2019; Zhang et al., 2020; Chiang et al., 2023) and curcumin (Tang et al., 2017; Voulgaropoulou et al., 2019; Shao et al., 2023; Abdul-Rahman et al., 2024), just to mention a few. These bioactive compounds are characterized by their anti-inflammatory, antioxidant effect, alongside other particular properties such as epigenetic regulation of key molecular pathways involved in AD. Finally, regarding supplements, vitamins B9 and B12 promote neuroprotection, improving synaptic plasticity (Mehrdad et al., 2023) and reducing neuroinflammation (Chen et al., 2021). It should be considered that high levels of homocysteine are considered a risk factor for AD (Miller, 1999; Seshadri et al., 2002). Vitamins B12 and B9 act as coenzymes for remethylation and posterior conversion to methionine (McCaddon and Miller, 2023), which would prevent hyperhomocysteinemia.

**FIGURE 3:**
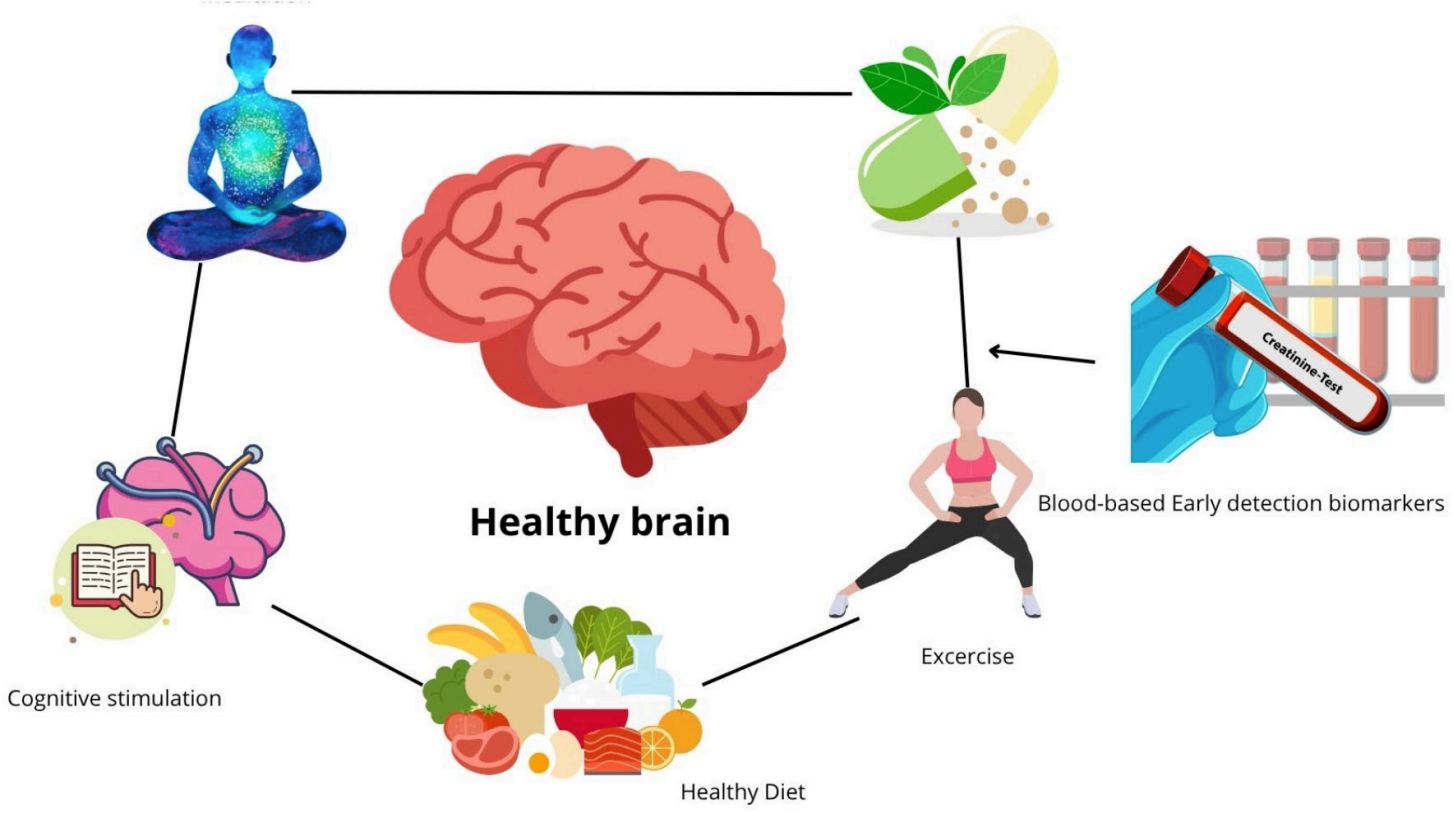
The five major scopes for prevention of Alzheimer’s disease. Considering that sporadic Alzheimer’s disease (AD) in over 80% can be prevented and its progression slowed by modifying some of the modifiable risk factors, nutrition, cognitive stimulation, a healthy diet, exercise, correct supplementation with nutraceuticals, and meditation all promote an improvement in cognitive performance and contribute to the prevention of AD. But for them to work, it is necessary to detect AD in a preclinical stage, prior to the manifestation of cognitive decline.

These major scopes for AD prevention/slow-down progression are summarized in Figure 3.

A recent United States POINTERS study demonstrated that with a structured lifestyle intervention, including MIND diet, regular moderate-to-high-intensity physical exercise, social engagement, cognitive challenge and cardiovascular health monitoring, a significant improvement in global cognition was observed (Baker et al., 2025). The latter includes at least 4 of the 5 scopes previously mentioned. Therefore, it is very relevant an early detection by sensitive biomarkers (Guzmán-Martínez et al., 2019), since it will allow an opportune intervention using clinically guided lifestyle changes.

## Conclusion

In this review, we briefly summarize the state-of-the-art regarding new frontiers in Alzheimer’s disease, from its etiopathogenesis to the most recent research in terms of effective therapies, biomarkers, and preventive measures. Given that even modest advances in therapeutic and preventative strategies that lead to small delays in the onset and progression of Alzheimer’s disease can significantly reduce the global burden of this disease (Brookmeyer et al., 2007), the development of effective interventions remains a high priority. The precise mechanisms underlying the pathogenesis of Alzheimer’s Disease are incompletely understood, but involve a complex interplay of genetic predisposition, environmental factors, and lifestyle influences.

However, for any treatment or preventive measure to be effective, it is necessary to screen patients in a pre-clinical stage, before the manifestation of the neuropsychological symptoms. Current FDA-approved biomarkers, such as PET scans and CSF biomarkers, only provide diagnosis in a post-clinical stage, when the neuropsychological symptoms are evident. Added to that, they are expensive and invasive, thus they are not a routine-based test that could be taken for preventive scopes. Now, current research is focused on providing a cost-effective, early-detection, blood-based biomarker. An example of this is the Alz-tau^®^ biomarker (Guzmán-Martínez et al., 2019), which is clinically validated and implemented in several health facilities. The challenge of blood-based biomarkers is that the proteins employed as biomarkers are in low quantity in blood or serum, which is why novel technologies such as SIMOA^®^ and Lumipulse^®^ provide ultrasensitive detection. However, implementing these technologies on a routine basis in clinical examination is still ongoing.

Since advances in terms of effective therapies and early-detection biomarkers are noted, but insufficient, prevention is key (Weninger et al., 2016; Guzman-Martinez et al., 2021a). Changes in lifestyle, such as adopting a Mediterranean diet, exercising, and reading to promote cognitive stimulation, may be key to preventing the cognitive decline associated with AD.

Further research is needed to identify and validate novel drug targets and to develop innovative therapeutic strategies that can effectively prevent, delay, or reverse the progression of this devastating disease.
